# Validation of the Spanish version of the hip outcome score: a multicenter study

**DOI:** 10.1186/1477-7525-12-70

**Published:** 2014-05-13

**Authors:** Roberto Seijas, Andrea Sallent, Miguel Angel Ruiz-Ibán, Oscar Ares, Oliver Marín-Peña, Ricardo Cuéllar, Alfonso Muriel

**Affiliations:** 1 Artroscopia GC - Fundación García Cugat, Hospital Quirón Barcelona (Spain), Pza. Alfonso Comín 5-7 Planta -1, 08023 Barcelona, Spain; 2 Basic Science Department, Universitat Internacional de Catalunya, Sant Cugat, Spain; 3 School of Medicine, Universitat de Barcelona (Spain), Barcelona, Spain; 4 Hospital Universitario Ramón y Cajal, Madrid, Spain; 5 Hospital Universitario Infanta Leonor, Madrid, Spain; 6 Hospital Universitario Donostia, San Sebastián, Spain; 7 IRYCIS (Instituto Ramón y Cajal de Investigación Sanitaria), Madrid, Spain; 8 CIBERESP (Centro de Investigación Biomédica en Red de Epidemiología y Salud Pública), Madrid, Spain

## Abstract

**Background:**

The Hip Outcome Score (HOS) is a self-reported questionnaire evaluating the outcomes of treatment interventions for hip pathologies, divided in 19 items of activities of daily life (ADL) and 9 sports’ items. The aim of the present study is to translate and validate HOS into Spanish.

**Methods:**

A prospective and multicenter study with 100 patients undergoing hip arthroscopy was performed between June 2012 and January 2013. Crosscultural adaptation was used to translate HOS into Spanish. Patients completed the questionnaire before and after surgery. Feasibility, reliability, internal consistency, construct validity (correlation with Western Ontario and McMaster Universities Osteoarthritis Index), ceiling and floor effects and sensitivity to change were assessed for the present study.

**Results:**

Mean age was 45.05 years old. 36 women and 64 men were included. *Feasibility:* 13% had at least one missing item within the ADL subscale and 17% within the sport subscale. *Reliability:* the translated version of HOS was highly reproducible with intraclass correlation coefficient of 0.95 for ADL and 0.94 for the sports subscale. *Internal* consistency was confirmed with Cronbach’s alpha >0.90 in both subscales. *Construct validity* showed statistically significant correlation with WOMAC. *Ceiling effect* was observed in 6% and 12% for ADL and sports subscale, respectively. *Floor effect* was found in 3% and 37% ADL and sports subscale, respectively. Large *sensitivity to change* was shown in both subscales.

**Conclusion:**

The translated version of HOS into Spanish has shown to be feasible, reliable and sensible to changes for patients undergoing hip arthroscopy. This validated translation of HOS allows for comparisons between studies involving either Spanish- or English-speaking patients.

**Level of evidence:**

Prognostic study, Level I

## Introduction

Since the description by Ganz et al. of femoroacetabular impingement (FAI) as a cause of hip osteoarthritis (OA) [1], especially in young adults, the goal of preventing this disabling disease has undoubtedly increased the indications of hip surgery in the young adult, especially hip arthroscopy [2]. Arthroscopic management of FAI has shown to be effective, with favorable outcomes altering the natural process of hip OA [3,4].

Questionnaires are a key tool in orthopedic surgery, as well as in other many specialties, in order to assess the impact that any procedure has on patients’ daily life. The classic tools designed to evaluate results in patients with hip pathology are less useful in young adults as they were initially designed to evaluate patients with OA and significant functional impairment, thus making them poor tools for assessing younger adults with subtle hip dysfunction that are however functionally significant.

The Hip Outcome Score (HOS) is a self-administered instrument divided into two subscales; activities-of-daily-living (ADL; 19 items) and sports (9 items), summing up a total of 28 items. It was developed by Martin, Kelly and Phillipon in 2006 in Pittsburgh, PA (USA) with the aim of evaluating the outcomes of treatment interventions for young patients with hip problems [5]. It has been validated for measuring outcomes following hip arthroscopy, remarking that the scale is specific for function assessment and not mental health [6,7]. A recent meta-analysis by Thorborg et al. suggest that the HOS may be the best available questionnaire for measuring hip arthroscopy outcomes [8].

The vast majority of these questionnaires have been developed in English, and therefore must undergo a validated translation that is mandatory for its use in a language different from the one in which it was developed [9-11]. Despite its increasing use, and perhaps owing to its relatively recent development, the HOS has only been translated to German [12]. The aim of this study is to translate the instrument and to validate the translation in order to provide physicians in all Spanish-speaking countries with a more specific evaluation tool for patients with hip disorders.

## Methods

The HOS questionnaire includes 19 questions about activities of daily living (ADL) and 9 sport-specific questions that offers a five difficulty-based response options, from “unable to do” to “no difficulty” to complete [5-7]. Two additional questions about the percentage of function in ADL and sports and another question regarding the “present functional level” are not included in the scoring. The ADL and sports subscale scores are normalized to obtain a range between 0-100, with higher scores representing better function. The HOS questionnaire can be scored if at least 14 items on the ADL subscale and 7 on the sports subscale have been completed [5].

### Crosscultural adaptation

The cross-cultural adaptation is a well-established protocol necessary to adapt health-related evaluation outcomes into other languages reaching excellent equivalence with the original form [13-16]. This process refers not only to translation, but also to the transcultural adaptation, adopting different lifestyles according to the different cultures, and can be summarized as follows:

1. Forward translation of the original Hip Outcome Score (English) into Spanish, by two independent professional translators (one English-native and one Spanish-native).

2. Review of the translations and synthesis of the first draft (version 0.1).

3. Back-translation of version 0.1 in Spanish to English by two English-native translators.

4. Review of both the back and forward translations. Drafting of the second version in Spanish (version 0.2) by an expert linguistic translator specialized in medical questionnaires and by a third translator.

5. Pretesting of the work (version 0.2) by a panel of 4 orthopedic physicians and 30 patients to assure that the text could be understood. Writing of version 1.0 (final version, see Additional file 1 for the final translated version of HOS to Spanish).

Patients included in the present study completed version 1.0 of the questionnaire and all statistical analysis of the psychometric parameters was performed upon this version 1.0.

### Patients

A prospective study with 100 patients was performed between June 2012 and January 2013 in order to carry out the transcultural adaptation and a validation of the Hip Outcome Score (HOS). Four surgeons recruited the patients’ series in four different centers.

Inclusion criteria for the patients were: age between 18 and 65 years old, presence of symptomatic hip pathology for at least 6 months that requires surgical treatment in the next two months but not earlier that 15 days from the present date, as well as having completed all questionnaires of both visits. Exclusion criteria were patient refusal to participate in the study. All patients were informed that data concerning their case would be further used for research and agreed to it. Oral and written informed consent was obtained from all of them. The Ethical Committee for Clinical Research (Comité Ético de Investigaciones Clínicas; CEIC) gave approval for the present study, which followed the guidance of the Declaration of Helsinki as adopted in 1964 and last revised in 2008. The patients were recruited consecutively between those attending the clinics of the participating surgeons; each surgeon recruited 25 patients.

The patients were given a questionnaire that included a copy of the translated HOS scale and a copy of the Spanish version of the Western Ontario and McMaster Universities Osteoarthritis Index (WOMAC) and were asked to fulfill it in clinic and were given a blank copy of the questionnaire with an stamped and addressed envelope with instructions to fulfill it again in 15 days and send it back to the investigators. Another copy of the questionnaire was fulfilled by the patients who been operated of their hip problems and were evaluated 6 months after the initial assessment. The WOMAC has been previously translated and validated in Spanish [11,17]. The WOMAC questionnaire evaluates pain, stiffness and function with five difficulty-based response options in patients with hip and/or knee OA [18]. Low scores appear in patients with a better quality of life, and vice versa. Therefore, an improvement is obtained when the overall score reduces (vice versa in the HOS questionnaire). Once the three subscales are added up, data was standardized to a range from 0 to 100 (being 0 the best health status and 100 the worst).

### Statistical analysis

Feasibility, reliability, internal consistency, construct validity (correlation with Western Ontario and McMaster Universities Osteoarthritis Index), ceiling and floor effects and sensitivity to change were assesses for the present study, in concordance with previous validation-related articles [10,12]. All statistical analysis was performed with SPSS statistical software version 21.0 (Chicago, IL, USA).

#### Feasibility

This parameter refers to the proportion of patients that did not answer any item, according to the preoperative visit. Feasibility was analyzed in the 100 questionnaires fulfilled in the first visit. The expected missing items proportions were similar to those obtained by the previous validated translation of the HOS to German; for the ADL subscale, 8/85 (9.4%) and 2/85 (2.4%) had 1 and 2 missing items, respectively. For the sports subscale, 14/85 (16.5%) with 1 missing item, 3/85 (3.5%) with 2 missing items and 1/85 (1.2%) with 3 missing items [12].

#### Reliability

A 15-day test-retest reliability was applied to the present manuscript. Of the 100 patients that fulfilled the initial translated version of HOS 80 sent back copies fulfilled 15 days after the initial evaluation. Of these, 14 were excluded as there was a difference of more than 5% in the reporting of the percentage of ADL or sports function between both questionnaires leaving 66 patients with two surveys fulfilled 15 days apart and with similar symptoms.

Test-retest reliability was determined using intraclass correlation coefficient (ICC) (two-way random effects model) [19] as well as standard error of measurement (SEM) and represented using a Bland-Altman plot. According to the previously published by Martin et al. [7], ICC scores were expected to be >0.90. In order to assess results, the minimal value considered acceptable for ICC was 0.75. Minimal detectable change (MDC) responded to the following formula: MDC = SEM × 1.4142 × 1.9 [12,20]. This expresses the degree of change required in an individual’s score in order to consider it as ‘real’ and not due to measurement errors. Ceiling and floor effects were analyzed in the 100 questionnaires fulfilled in the preoperative visit.

#### Internal consistency

Cronbach’s α is used to measure internal consistency and a questionnaire is usually considered as consistent when α >0.8 [21]. Internal consistency was analyzed in the 100 questionnaires fulfilled in the first visit.

#### Construct validity

Defined as the degree to which an instrument measures the characteristic being investigated. This was measured comparing the results obtained in the 100 questionnaires fulfilled in the first visit in both scales HOS and WOMAC. Construct validity was assessed with a correlation analysis between both scales using the Spearman’s Rho. A threshold of r > 0.5 is considered acceptable suggesting moderate to high correlation [21]. WOMAC values were first reversed as these two scales are orientated in opposite directions in order to obtain positive values.

#### Ceiling and floor effects

The ceiling effect refers to the percentage of patients with maximum score within the questionnaire, indicating the best clinical outcome. On the other hand, the floor effect accounts for the proportion of patients with a minimum score, showing the worst clinical outcome. Ceiling and floor effects can be worked out as percentage of patients with maximum or minimum scores, respectively, or either with the maximum score (100 points in this case) minus the minimal detectable change (MDC) and worst score (0 points) plus the MDC, respectively. Within the present manuscript, both methods were used to describe these effects.

#### Sensitivity to change

A total of 78 patients were available for evaluation with the questionnaire after surgery and 6 months after the initial evaluation. The differences in mean scores before and after surgery at 6 months postoperative, using paired *t*-test or Wilcoxon signed-ranked test using an analysis for homogeneous samples with homogeneous expected change [22]. The ability of an instrument to detect change is quantified dividing the mean change by the standard deviation in change: the standardized response mean (SRM) [23]. SRM values of 0.20, 0.50 and 0.80 represent small, moderate and large sensitivity to change, respectively [24].

## Results

A total of 36 women and 64 men with a mean age was 45.1 years old (SD 12.1, range 18 to 65 years) were included in the study. Clinical diagnosis was as follows: 37 FAI (combined impingement), 26 FAI (Cam-type lesion), 15 combined Cam and labrum, 5 Tönnis II, 5 Tönnis 1, 4 FAI (Pincer-type lesion), 3 labrum, 2 trochanteritis, 1 slipped capital femoral epiphysiolysis sequelae, 1 Perthes sequelae and 1 osteonecrosis.

### Cross-cultural adaptation

Forward and back-translation revealed no major problems with language or grammatical errors. Small discrepancies rose for synonyms; “getting in and out of an average car”, where average was translated to “estándar”, later to “normal”; light to moderate work, was translated to “leve y moderado”, finally to “ligero y moderado”. Pre-testing of the final version 1.0 revealed no further complications within comprehension.

### Feasibility

One hundred questionnaires were studied for feasibility. 87 patients (87%) filled out the ADL subscale completely. On the other hand, 83 patients (83%) answered all questions of the sport subscale. No questionnaire was registered with three or more missing items either in the ADL or sports subscale. Thus, the total subscale score could be calculated in all cases (Table 1).

**Table 1:** Feasibility of the hip outcome score; number of missing items registered within our questionnaires

	**1 missing item**	**2 missing items**
**Activities of daily life subscale**	11 (11%)	2 (2%)
**Sports subscale**	16 (16%)	1 (1%)

### Reliability

**Figure 1:**
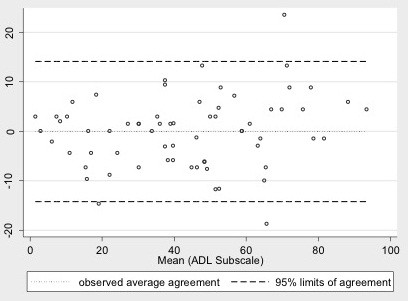
**Bland-Altman plot illustrating agreement between repeated measurements of activities of daily life (ADL) subscale.** The difference between measurements is shown against the average Hip Outcome Score-ADL subscale score of the 100 participants. The three horizontal lines indicate the mean individual differences ± 1.96 standard deviation (limits of agreement). Mean difference -0.05, 95% limits of agreement = -14.2:14.1.

**Figure 2:**
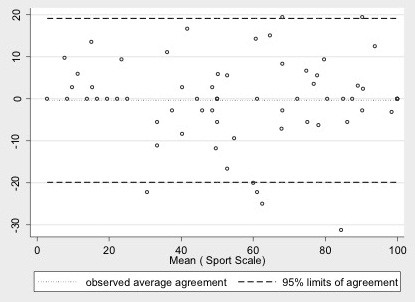
**Bland-Altman plot illustrating agreement between repeated measurements of the sport subscale.** The difference between measurements is shown against the average Hip Outcome Score-sport subscale of the 100 participants. The three horizontal lines indicate the mean individual differences ± 1.96 standard deviation (limits of agreement). Mean difference -0.3, 95% limits of agreement = -19.8:19.2.

Both subscales (ADL and sports) obtained excellent ICC within the 15-day test-retest reliability; 0.95 (CI 95%, 0.92; 0.97) for the ADL subscale and 0.94 (CI 95%, 0.89; 0.97) for the sports subscale. Mean scores for the ADL subscale at test and retest were 43.3 points (SD 24) and 43.2 points (SD 22), respectively. The sports subscale had a mean score of 55.6 points (SD 28) and 56 points (SD 28) for the test and retest respectively. The SEM was ± 5.1 for the ADL subscale and ±8.5 for the sports subscale. Thus, MDC was 13.7 points within the ADL subscale and 22.8 points for the sports subscale (Figures 1 and 2).

### Internal consistency

Cronbach’s α of 0.95 for the ADL subscale and 0.9 for the sport subscale confirmed a high internal consistency.

### Construct validity

HOS-ADL showed excellent construct validity against the WOMAC score (Table 2) in all subscales, being statistically significant. HOS-sports showed excellent validity when assessed against the pain and function WOMAC scores.

**Table 2:** Construct validity showing the correlations between Hip Outcome Score (HOS) and WOMAC questionnaires

		**HOS**	
		**ADL**	**Sports**
**WOMAC**	**Pain**	0.699	0.607
	**Stiffness**	0.667	0.499
	**Function**	0.778	0.764

All correlations were statistically significant at a p value <0.001. ADL: activities of daily life subscale.

### Ceiling and floor effects

Taking into account the MDC, ceiling effect was observed in 6% of patients whereas floor effect accounted for 3% of patients, accounting for the ADL subscale. The sport subscale showed ceiling effect in 12% of cases and floor effect in 37% of cases. When only the best (100 points) and worst (0 point) scores were considered, there would be no floor effect on either subscale and 1 ceiling effect on the sports subscale but no ceiling effect within the ADL subscale.

### Sensitivity to change

SRM scores within the ADL subscale were 1.53 and 1.27 for the sports subscale, showing large sensitivity to change.

## Discussion

The present study aimed to translate and validate the Hip Outcome Score (HOS) to Spanish. Given the abovementioned results, a correct cross-cultural adaptation and posterior validation has been proven, showing that the HOS questionnaire can be used in Spanish-speaking countries.

Health-related questionnaires are a means of quantifying a subjective experience, aiming to provide the professional with patients’ satisfaction and quality of life information following surgical or nonsurgical treatments. WOMAC questionnaire is currently the only validated and hip-specific questionnaire in Spanish available for surgeons treating younger active patients with hip problems in Spanish speaking countries [11], whereas English-speaking countries enjoy of more validated questionnaires and scores. This study has allowed for the development of such a tool.

The feasibility of the score was generally excellent but, out of the 17 patients with one or two missing items within the sport subscale, 13 had left blank item 3 (“swinging objects like a golf club”). This observation can be explained by the fact that golf is not a so-popular sport in Spain, and although the question asks for the movement as in golf swing, patients may have left this question blank misunderstanding the movement. Replacing the ‘golf’ item with a different sport was considered for the Spanish population, but finally the question was left since there was no other alternative that presented more popularity and the same hip demand. It was observed that the sports subscale presented more missing items than the ADL subscale, which can be explained by the fact that patients can more easily answer questions about activities that are performed daily rather than about sports that they might have never practiced.

The questionnaire showed an excellent reliability with ICC scores of 0.95 and 0.94 for the ADL and sports subscale, respectively, in line with the previously published by the original authors (0.98 and 0.92 respectively) [7], as well as by the German translation authors (0.94 and 0.89, respectively) [12]. As for the ceiling and floor effects, this were also in accordance with both Martin’s and Naal’s previous studies [7,12]. Martin et al. showed only 1 patient who scored 100% in both the ADL and sport subscale in the preoperative visit (considering only the best possible scores), in line with the present study, with only 1 patient who scored 100% in the sports subscale (and none for the ADL subscale) [7]. As the previously mentioned work by Naal et al., ceiling effect was higher than the floor effect in the ADL subscale and vice versa for the sport subscale, in consonance with the present study [12].

The present paper provides support for the concurrent construct validity of the scale, comparing HOS and WOMAC, given the strength of correlations (all >0.5). Martin et al. showed a strong correlation between HOS and the SF-36 physical function and physical component subscale (0.76 and 0.74 respectively for the HOS-ADL subscale and 0.72 and 0.68 for the HOS-sports subscale), as expected the correlation with the SF-36 mental components was weaker [5]. Equally, the German study by Naal et al. showed an excellent correlation between WOMAC and HOS [12], in line with what can be observed in the present study.

Internal consistency, through Cronbach’s α was also corroborated with scores over 0.8 for both subscales, as it had been hypothesized [21].

The scale improved in ADL and sports subscale with a large sensitivity to change given the SRM results obtained.

The present study includes 100 patients, fifteen more than the previous translation of HOS into German [12], as well as evaluating the same metric properties as in the German translation in addition to sensitivity to change, as assessed in previously validated translations [10,22].

### Limitations

Although ours was a multicenter study, all hospitals involved were located in Spain. Thus, some words of the translated version should be reviewed when administering the questionnaire in other Spanish-speaking countries such as South America, for example, regarding the word car, whereas in Spain it is translated as “coche”, South-American countries use the word “auto”. Despite having an official organization that regulates the Spanish language (Real Academia Española), local colloquialisms are extraordinarily frequent due to the extensive geographic distribution of the Spanish language and the high number of available words.

Secondly, only four Spanish hospitals were included in the collection of data, although more hospitals would be better and represent a wider distribution. However, the socioeconomic and cultural levels were widely represented within these hospitals (combining private practice, cosmopolitan public hospitals and smaller regional hospital, as well as populations form both urban or rural areas).

Third, responsiveness to clinical change is another important criterion to be measured for a translated questionnaire in order to assess if HOS is more sensitive and specific than other existing instruments. Martin et al. have already proven an excellent responsiveness for the original English version but it was not performed in this study and should be performed in future studies, as the HOS is in fact more sensitive than the WOMAC questionnaire in this population [7].

Fourth, questionnaires are a means of quantifying a subjective experience. However, despite HOS is yet not stratified, as for example Harris Hip Score [25], it is divided into two separate scales (ADL and sports) in order to evaluate the impact in quality of life of each subscale.

Last, a greater number of patients could have been collected for the present study, however, according to previous papers, our group of patients was greater than any previous.

In conclusion, the presented Spanish version of the HOS questionnaire provides strong evidence that the HOS is a tool with valid construct, reliable, feasible and with large sensitivity to change and internal consistency for the measurement of patient-orientated outcomes regarding hip disorders in the young adult. The present validation of the HOS allows new comparisons between Spanish-speaking patients and those with already validated questionnaires (e.g. English, German).

## Competing interests

The authors declare that they have no competing interest.

## Authors’ contribution

RS participated in acquisition of patients’ data, supervised the draft and contributed to the conception and design of the study. AS participated in drafting the article, analysis of results and designed the manuscript. MARI participated in acquisition of patients’ data, analysis of data and supervision of the manuscript. RC participated in acquisition of patients’ data, interpretation of results and supervision of the manuscript. OMP participated in acquisition of patients’ data, contributed to conception and design and revision of results. OA participated in drafting the article, analysis and interpretation of data. AM was in charge of the statistical analysis and revised the manuscript. All authors read and approved the final version of the manuscript.

## Supplementary Material

Additional file 1 Escala de cadera para las actividades de la vida diaria. Click here for file
