# Supplementary material for: Validation of the Spanish version of the hip outcome score: a multicenter study
Source: Health Qual Life Outcomes. 2014 May 13;12:70. doi: 10.1186/1477-7525-12-70 (PMC4064819; doi:10.1186/1477-7525-12-70)
Supplement: Additional file 1 — Escala de cadera para las actividades de la vida diaria. [file 1477-7525-12-70-S1.pdf]

## <u>ESCALA DE CADERA PARA LAS ACTIVIDADES DE LA VIDA DIARIA.</u>

RESPONDA **TODAS LAS PREGUNTAS** CON **UNA SOLA RESPUESTA** LA QUE CORRESPONDA A SU SITUACIÓN EN LOS ÚLTIMOS DÍAS.
SI HA HABIDO ALGUNA COSA QUE NO HA PODIDO REALIZAR DEBIDO A OTRAS CAUSAS DIFERENTES A SU CADERA MARQUE **NA.**

| | Ninguna dificultad | Dificultad leve | Dificultad Moderada | Dificultad extrema | Incapaz de hacerlo | No Responde |
|---|---|---|---|---|---|---|
| Estar de pie durante 15 minutos | ☐ | ☐ | ☐ | ☐ | ☐ | ☐ |
| Entrar y salir de un coche normal | ☐ | ☐ | ☐ | ☐ | ☐ | ☐ |
| Ponerse calcetines y zapatos | ☐ | ☐ | ☐ | ☐ | ☐ | ☐ |
| Subir caminando por terrenos empinados | ☐ | ☐ | ☐ | ☐ | ☐ | ☐ |
| Bajar caminando por terrenos empinados | ☐ | ☐ | ☐ | ☐ | ☐ | ☐ |
| Subir un tramo de escaleras | ☐ | ☐ | ☐ | ☐ | ☐ | ☐ |
| Bajar un tramo de escaleras | ☐ | ☐ | ☐ | ☐ | ☐ | ☐ |
| Subir y bajar los bordillos de las aceras | ☐ | ☐ | ☐ | ☐ | ☐ | ☐ |
| Ponerse en cuclillas agachándose lo máximo posible | ☐ | ☐ | ☐ | ☐ | ☐ | ☐ |
| Entrar y salir de la bañera | ☐ | ☐ | ☐ | ☐ | ☐ | ☐ |
| Estar sentado durante 15 minutos | ☐ | ☐ | ☐ | ☐ | ☐ | ☐ |
| Empezar a caminar | ☐ | ☐ | ☐ | ☐ | ☐ | ☐ |
| Caminar durante 10 minutos aproximadamente | ☐ | ☐ | ☐ | ☐ | ☐ | ☐ |
| Caminar durante 15 minutos o más. | ☐ | ☐ | ☐ | ☐ | ☐ | ☐ |

A causa de su cadera, ¿qué grado de dificultad tiene para realizar las actividades siguientes?

| | Ninguna dificultad | Dificultad leve | Dificultad Moderada | Dificultad extrema | Incapaz de hacerlo | No Responde |
|---|---|---|---|---|---|---|
| Girar/pivotar sobre la pierna afectada | ☐ | ☐ | ☐ | ☐ | ☐ | ☐ |
| Darse la vuelta en la cama | ☐ | ☐ | ☐ | ☐ | ☐ | ☐ |
| Efectuar trabajos ligeros o moderados (estar de pie, caminar) | ☐ | ☐ | ☐ | ☐ | ☐ | ☐ |
| Efectuar trabajos pesados (empujar/arrastrar, subirse a sitios, cargar pesos) | ☐ | ☐ | ☐ | ☐ | ☐ | ☐ |
| Realizar actividades de ocio | ☐ | ☐ | ☐ | ☐ | ☐ | ☐ |

¿Como puntuaría usted de 0 a 100 su nivel actual de funcionamiento durante sus actividades de la vida diaria habituales (siendo 100 el nivel de funcionamiento antes de su problema de cadera y 0 la incapacidad para realizar cualquiera de sus actividades de la vida diaria habituales)?

☐☐☐.0%

Subescala de deportes

A causa de su cadera, ¿qué grado de dificultad tiene para realizar las actividades siguientes?

| | Ninguna dificultad | Dificultad leve | Dificultad Moderada | Dificultad extrema | Incapaz de hacerlo | No Responde |
|---|---|---|---|---|---|---|
| Correr 1,5 km | ☐ | ☐ | ☐ | ☐ | ☐ | ☐ |
| Saltar | ☐ | ☐ | ☐ | ☐ | ☐ | ☐ |
| Balancear un objeto, como un palo de golf | ☐ | ☐ | ☐ | ☐ | ☐ | ☐ |
| Caer sobre los pies tras un salto | ☐ | ☐ | ☐ | ☐ | ☐ | ☐ |
| Echar a correr y pararse rápidamente | ☐ | ☐ | ☐ | ☐ | ☐ | ☐ |
| Efectuar cambios de dirección / movimientos laterales | ☐ | ☐ | ☐ | ☐ | ☐ | ☐ |
| Efectuar actividades de bajo impacto como caminar deprisa | ☐ | ☐ | ☐ | ☐ | ☐ | ☐ |
| Capacidad para ejecutar la actividad con su técnica habitual | ☐ | ☐ | ☐ | ☐ | ☐ | ☐ |
| Capacidad para practicar el deporte que desee durante el tiempo que quiera | ☐ | ☐ | ☐ | ☐ | ☐ | ☐ |

¿Cómo puntuaría de 0 a 100 su nivel actual de funcionamiento en las actividades relacionadas con el deporte (siendo 100 su nivel de funcionamiento antes de su problema de cadera y 0 la incapacidad para realizar cualquiera de sus actividades de la vida diaria habituales)?

☐☐☐.0%

¿Cómo puntuaría su nivel actual de funcionamiento?

Normal    Casi Normal    Anormal    Muy anormal
